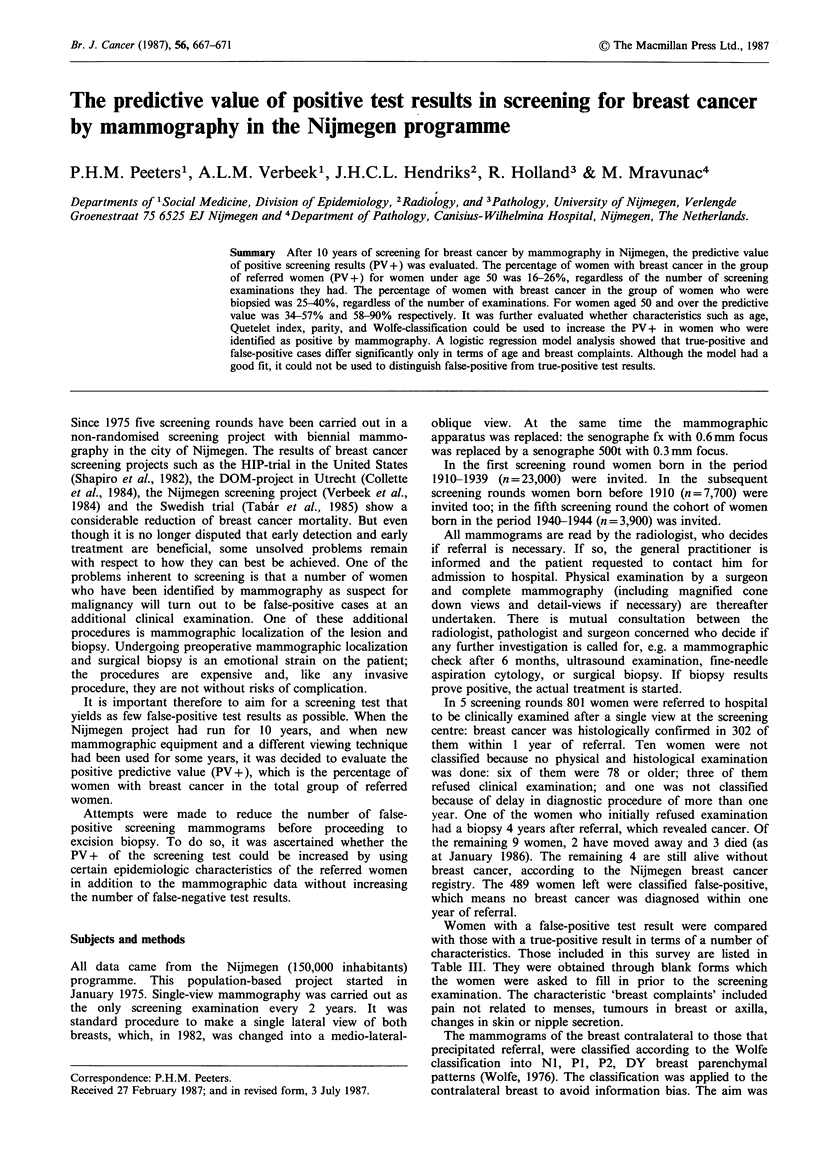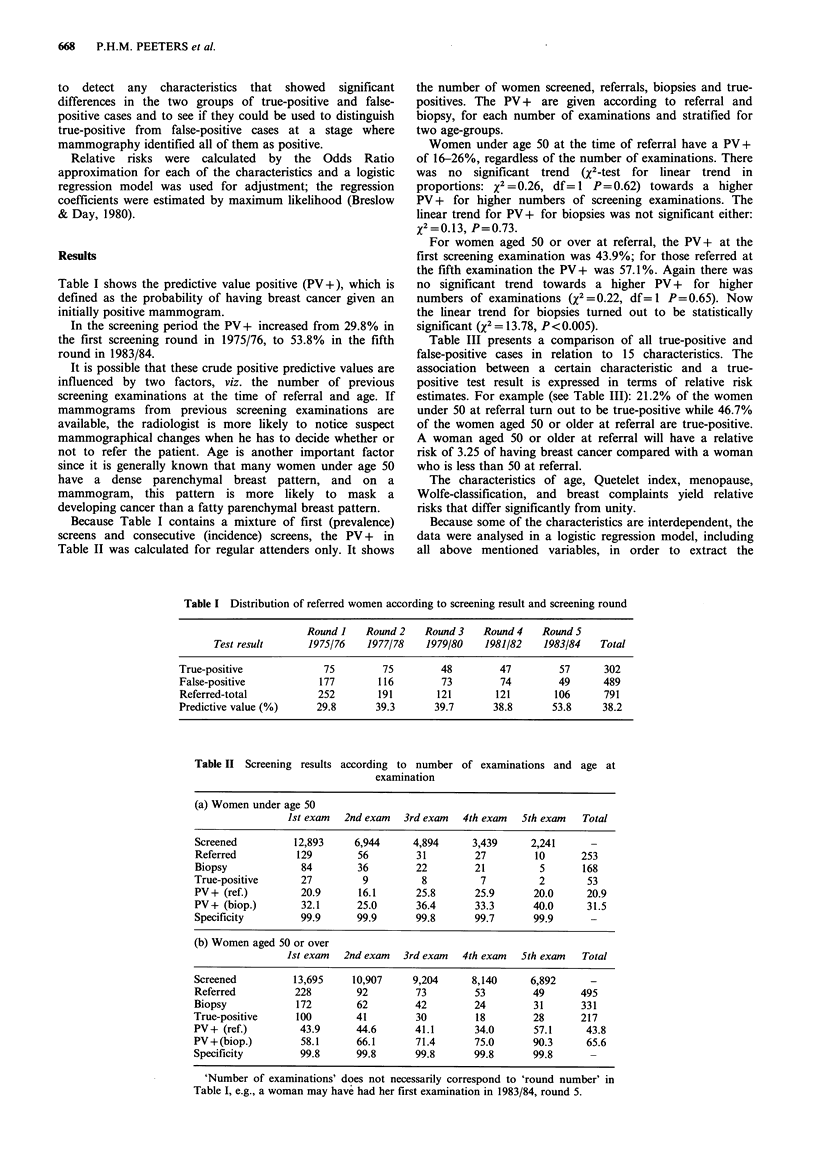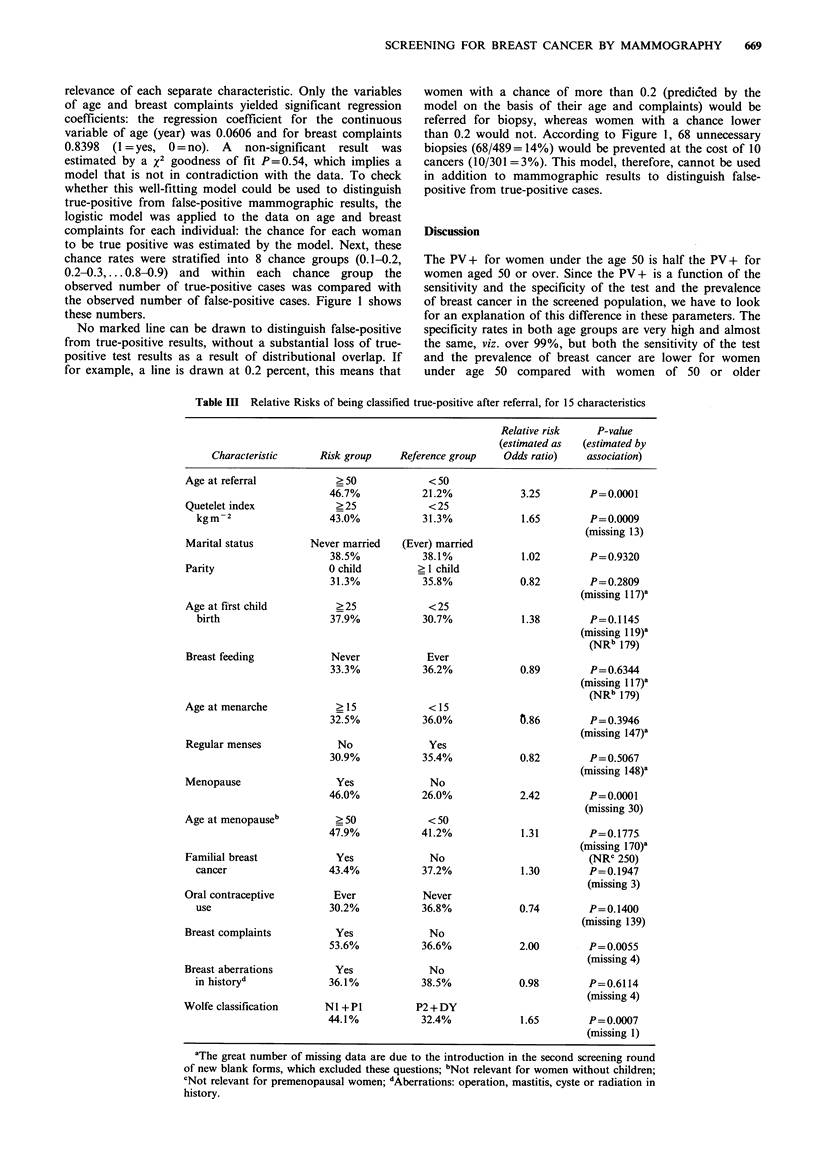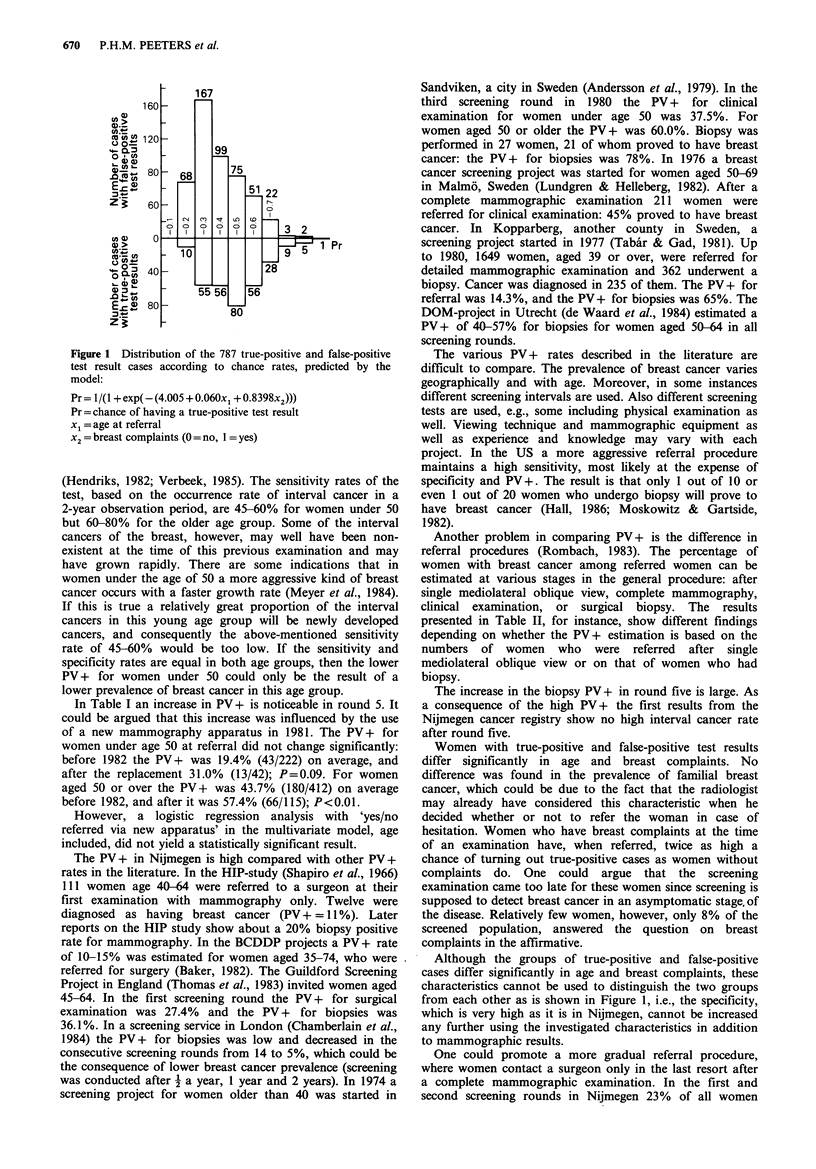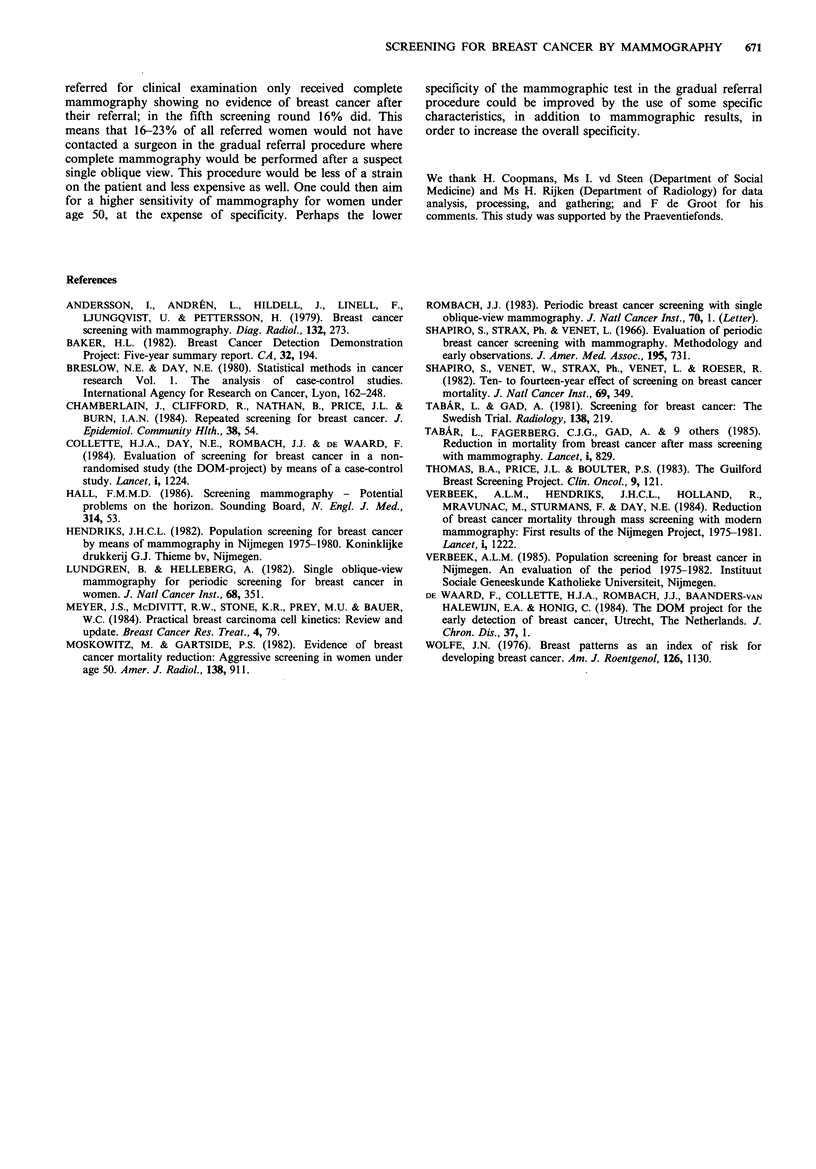# The predictive value of positive test results in screening for breast cancer by mammography in the Nijmegen programme.

**DOI:** 10.1038/bjc.1987.263

**Published:** 1987-11

**Authors:** P. H. Peeters, A. L. Verbeek, J. H. Hendriks, R. Holland, M. Mravunac

**Affiliations:** Department of Social Medicine, University of Nijmegen, The Netherlands.

## Abstract

After 10 years of screening for breast cancer by mammography in Nijmegen, the predictive value of positive screening results (PV+) was evaluated. The percentage of women with breast cancer in the group of referred women (PV+) for women under age 50 was 16-26%, regardless of the number of screening examinations they had. The percentage of women with breast cancer in the group of women who were biopsied was 25-40%, regardless of the number of examinations. For women aged 50 and over the predictive value was 34-57% and 58-90% respectively. It was further evaluated whether characteristics such as age, Quetelet index, parity, and Wolfe-classification could be used to increase the PV+ in women who were identified as positive by mammography. A logistic regression model analysis showed that true-positive and false-positive cases differ significantly only in terms of age and breast complaints. Although the model had a good fit, it could not be used to distinguish false-positive from true-positive test results.


*Br. J. Cancer* (1987), **56**, 667–671 © The Macmillan Press Ltd., 1987

### The predictive value of positive test results in screening for breast cancer by mammography in the Nijmegen programme

P.H.M. Peeters[1], A.L.M. Verbeek[1], J.H.C.L. Hendriks[2], R. Holland[3] & M. Mravunac[4]

*Departments of [1]Social Medicine, Division of Epidemiology, [2]Radiology, and [3]Pathology, University of Nijmegen, Verlengde Groenestraat 75 6525 EJ Nijmegen and [4]Department of Pathology, Canisius-Wilhelmina Hospital, Nijmegen, The Netherlands.*

**Summary** After 10 years of screening for breast cancer by mammography in Nijmegen, the predictive value of positive screening results (PV+) was evaluated. The percentage of women with breast cancer in the group of referred women (PV+) for women under age 50 was 16–26%, regardless of the number of screening examinations they had. The percentage of women with breast cancer in the group of women who were biopsied was 25–40%, regardless of the number of examinations. For women aged 50 and over the predictive value was 34–57% and 58–90% respectively. It was further evaluated whether characteristics such as age, Quetelet index, parity, and Wolfe-classification could be used to increase the PV+ in women who were identified as positive by mammography. A logistic regression model analysis showed that true-positive and false-positive cases differ significantly only in terms of age and breast complaints. Although the model had a good fit, it could not be used to distinguish false-positive from true-positive test results.

Since 1975 five screening rounds have been carried out in a non-randomised screening project with biennial mammography in the city of Nijmegen. The results of breast cancer screening projects such as the HIP-trial in the United States (Shapiro *et al.*, 1982), the DOM-project in Utrecht (Collette *et al.*, 1984), the Nijmegen screening project (Verbeek *et al.*, 1984) and the Swedish trial (Tabár *et al.*, 1985) show a considerable reduction of breast cancer mortality. But even though it is no longer disputed that early detection and early treatment are beneficial, some unsolved problems remain with respect to how they can best be achieved. One of the problems inherent to screening is that a number of women who have been identified by mammography as suspect for malignancy will turn out to be false-positive cases at an additional clinical examination. One of these additional procedures is mammographic localization of the lesion and biopsy. Undergoing preoperative mammographic localization and surgical biopsy is an emotional strain on the patient; the procedures are expensive and, like any invasive procedure, they are not without risks of complication.

It is important therefore to aim for a screening test that yields as few false-positive test results as possible. When the Nijmegen project had run for 10 years, and when new mammographic equipment and a different viewing technique had been used for some years, it was decided to evaluate the positive predictive value (PV+), which is the percentage of women with breast cancer in the total group of referred women.

Attempts were made to reduce the number of false-positive screening mammograms before proceeding to excision biopsy. To do so, it was ascertained whether the PV+ of the screening test could be increased by using certain epidemiologic characteristics of the referred women in addition to the mammographic data without increasing the number of false-negative test results.

### Subjects and methods

All data came from the Nijmegen (150,000 inhabitants) programme. This population-based project started in January 1975. Single-view mammography was carried out as the only screening examination every 2 years. It was standard procedure to make a single lateral view of both breasts, which, in 1982, was changed into a medio-lateral-oblique view. At the same time the mammographic apparatus was replaced: the senographe fx with 0.6 mm focus was replaced by a senographe 500t with 0.3 mm focus.

In the first screening round women born in the period 1910–1939 ($n=23,000$) were invited. In the subsequent screening rounds women born before 1910 ($n=7,700$) were invited too; in the fifth screening round the cohort of women born in the period 1940–1944 ($n=3,900$) was invited.

All mammograms are read by the radiologist, who decides if referral is necessary. If so, the general practitioner is informed and the patient requested to contact him for admission to hospital. Physical examination by a surgeon and complete mammography (including magnified cone down views and detail-views if necessary) are thereafter undertaken. There is mutual consultation between the radiologist, pathologist and surgeon concerned who decide if any further investigation is called for, e.g. a mammographic check after 6 months, ultrasound examination, fine-needle aspiration cytology, or surgical biopsy. If biopsy results prove positive, the actual treatment is started.

In 5 screening rounds 801 women were referred to hospital to be clinically examined after a single view at the screening centre: breast cancer was histologically confirmed in 302 of them within 1 year of referral. Ten women were not classified because no physical and histological examination was done: six of them were 78 or older; three of them refused clinical examination; and one was not classified because of delay in diagnostic procedure of more than one year. One of the women who initially refused examination had a biopsy 4 years after referral, which revealed cancer. Of the remaining 9 women, 2 have moved away and 3 died (as at January 1986). The remaining 4 are still alive without breast cancer, according to the Nijmegen breast cancer registry. The 489 women left were classified false-positive, which means no breast cancer was diagnosed within one year of referral.

Women with a false-positive test result were compared with those with a true-positive result in terms of a number of characteristics. Those included in this survey are listed in Table III. They were obtained through blank forms which the women were asked to fill in prior to the screening examination. The characteristic 'breast complaints' included pain not related to menses, tumours in breast or axilla, changes in skin or nipple secretion.

The mammograms of the breast contralateral to those that precipitated referral, were classified according to the Wolfe classification into N1, P1, P2, DY breast parenchymal patterns (Wolfe, 1976). The classification was applied to the contralateral breast to avoid information bias. The aim was

Correspondence: P.H.M. Peeters.
Received 27 February 1987; and in revised form, 3 July 1987.

to detect any characteristics that showed significant differences in the two groups of true-positive and false-positive cases and to see if they could be used to distinguish true-positive from false-positive cases at a stage where mammography identified all of them as positive.

Relative risks were calculated by the Odds Ratio approximation for each of the characteristics and a logistic regression model was used for adjustment; the regression coefficients were estimated by maximum likelihood (Breslow & Day, 1980).

### Results

Table I shows the predictive value positive (PV+), which is defined as the probability of having breast cancer given an initially positive mammogram.

In the screening period the PV+ increased from 29.8% in the first screening round in 1975/76, to 53.8% in the fifth round in 1983/84.

It is possible that these crude positive predictive values are influenced by two factors, *viz.* the number of previous screening examinations at the time of referral and age. If mammograms from previous screening examinations are available, the radiologist is more likely to notice suspect mammographical changes when he has to decide whether or not to refer the patient. Age is another important factor since it is generally known that many women under age 50 have a dense parenchymal breast pattern, and on a mammogram, this pattern is more likely to mask a developing cancer than a fatty parenchymal breast pattern.

Because Table I contains a mixture of first (prevalence) screens and consecutive (incidence) screens, the PV+ in Table II was calculated for regular attenders only. It shows the number of women screened, referrals, biopsies and true-positives. The PV+ are given according to referral and biopsy, for each number of examinations and stratified for two age-groups.

Women under age 50 at the time of referral have a PV+ of 16–26%, regardless of the number of examinations. There was no significant trend ($\chi^2$-test for linear trend in proportions: $\chi^2=0.26$, df=1 $P=0.62$) towards a higher PV+ for higher numbers of screening examinations. The linear trend for PV+ for biopsies was not significant either: $\chi^2=0.13$, $P=0.73$.

For women aged 50 or over at referral, the PV+ at the first screening examination was 43.9%; for those referred at the fifth examination the PV+ was 57.1%. Again there was no significant trend towards a higher PV+ for higher numbers of examinations ($\chi^2=0.22$, df=1 $P=0.65$). Now the linear trend for biopsies turned out to be statistically significant ($\chi^2=13.78$, $P<0.005$).

Table III presents a comparison of all true-positive and false-positive cases in relation to 15 characteristics. The association between a certain characteristic and a true-positive test result is expressed in terms of relative risk estimates. For example (see Table III): 21.2% of the women under 50 at referral turn out to be true-positive while 46.7% of the women aged 50 or older at referral are true-positive. A woman aged 50 or older at referral will have a relative risk of 3.25 of having breast cancer compared with a woman who is less than 50 at referral.

The characteristics of age, Quetelet index, menopause, Wolfe-classification, and breast complaints yield relative risks that differ significantly from unity.

Because some of the characteristics are interdependent, the data were analysed in a logistic regression model, including all above mentioned variables, in order to extract the

**Table I** Distribution of referred women according to screening result and screening round

| Test result | *Round 1 1975/76* | *Round 2 1977/78* | *Round 3 1979/80* | *Round 4 1981/82* | *Round 5 1983/84* | *Total* |
|---|---|---|---|---|---|---|
| True-positive | 75 | 75 | 48 | 47 | 57 | 302 |
| False-positive | 177 | 116 | 73 | 74 | 49 | 489 |
| Referred-total | 252 | 191 | 121 | 121 | 106 | 791 |
| Predictive value (%) | 29.8 | 39.3 | 39.7 | 38.8 | 53.8 | 38.2 |

**Table II** Screening results according to number of examinations and age at examination

| (a) Women under age 50 | *1st exam* | *2nd exam* | *3rd exam* | *4th exam* | *5th exam* | *Total* |
|---|---|---|---|---|---|---|
| Screened | 12,893 | 6,944 | 4,894 | 3,439 | 2,241 | – |
| Referred | 129 | 56 | 31 | 27 | 10 | 253 |
| Biopsy | 84 | 36 | 22 | 21 | 5 | 168 |
| True-positive | 27 | 9 | 8 | 7 | 2 | 53 |
| PV+ (ref.) | 20.9 | 16.1 | 25.8 | 25.9 | 20.0 | 20.9 |
| PV+ (biop.) | 32.1 | 25.0 | 36.4 | 33.3 | 40.0 | 31.5 |
| Specificity | 99.9 | 99.9 | 99.8 | 99.7 | 99.9 | – |
| (b) Women aged 50 or over | *1st exam* | *2nd exam* | *3rd exam* | *4th exam* | *5th exam* | *Total* |
| Screened | 13,695 | 10,907 | 9,204 | 8,140 | 6,892 | – |
| Referred | 228 | 92 | 73 | 53 | 49 | 495 |
| Biopsy | 172 | 62 | 42 | 24 | 31 | 331 |
| True-positive | 100 | 41 | 30 | 18 | 28 | 217 |
| PV+ (ref.) | 43.9 | 44.6 | 41.1 | 34.0 | 57.1 | 43.8 |
| PV+(biop.) | 58.1 | 66.1 | 71.4 | 75.0 | 90.3 | 65.6 |
| Specificity | 99.8 | 99.8 | 99.8 | 99.8 | 99.8 | – |

'Number of examinations' does not necessarily correspond to 'round number' in Table I, e.g., a woman may have had her first examination in 1983/84, round 5.

relevance of each separate characteristic. Only the variables of age and breast complaints yielded significant regression coefficients: the regression coefficient for the continuous variable of age (year) was 0.0606 and for breast complaints 0.8398 (1 = yes, 0 = no). A non-significant result was estimated by a $\chi^2$ goodness of fit $P = 0.54$, which implies a model that is not in contradiction with the data. To check whether this well-fitting model could be used to distinguish true-positive from false-positive mammographic results, the logistic model was applied to the data on age and breast complaints for each individual: the chance for each woman to be true positive was estimated by the model. Next, these chance rates were stratified into 8 chance groups (0.1–0.2, 0.2–0.3,...0.8–0.9) and within each chance group the observed number of true-positive cases was compared with the observed number of false-positive cases. Figure 1 shows these numbers.

No marked line can be drawn to distinguish false-positive from true-positive results, without a substantial loss of true-positive test results as a result of distributional overlap. If for example, a line is drawn at 0.2 percent, this means that women with a chance of more than 0.2 (predicted by the model on the basis of their age and complaints) would be referred for biopsy, whereas women with a chance lower than 0.2 would not. According to Figure 1, 68 unnecessary biopsies (68/489 = 14%) would be prevented at the cost of 10 cancers (10/301 = 3%). This model, therefore, cannot be used in addition to mammographic results to distinguish false-positive from true-positive cases.

### Discussion

The PV+ for women under the age 50 is half the PV+ for women aged 50 or over. Since the PV+ is a function of the sensitivity and the specificity of the test and the prevalence of breast cancer in the screened population, we have to look for an explanation of this difference in these parameters. The specificity rates in both age groups are very high and almost the same, *viz.* over 99%, but both the sensitivity of the test and the prevalence of breast cancer are lower for women under age 50 compared with women of 50 or older

**Table III** Relative Risks of being classified true-positive after referral, for 15 characteristics

| *Characteristic* | *Risk group* | *Reference group* | *Relative risk (estimated as Odds ratio)* | *P-value (estimated by association)* |
|---|---|---|---|---|
| Age at referral | ≧50<br>46.7% | <50<br>21.2% | 3.25 | $P = 0.0001$ |
| Quetelet index kg m$^{-2}$ | ≧25<br>43.0% | <25<br>31.3% | 1.65 | $P = 0.0009$ (missing 13) |
| Marital status | Never married<br>38.5% | (Ever) married<br>38.1% | 1.02 | $P = 0.9320$ |
| Parity | 0 child<br>31.3% | ≧1 child<br>35.8% | 0.82 | $P = 0.2809$ (missing 117)[a] |
| Age at first child birth | ≧25<br>37.9% | <25<br>30.7% | 1.38 | $P = 0.1145$ (missing 119)[a] (NR[b] 179) |
| Breast feeding | Never<br>33.3% | Ever<br>36.2% | 0.89 | $P = 0.6344$ (missing 117)[a] (NR[b] 179) |
| Age at menarche | ≧15<br>32.5% | <15<br>36.0% | 0.86 | $P = 0.3946$ (missing 147)[a] |
| Regular menses | No<br>30.9% | Yes<br>35.4% | 0.82 | $P = 0.5067$ (missing 148)[a] |
| Menopause | Yes<br>46.0% | No<br>26.0% | 2.42 | $P = 0.0001$ (missing 30) |
| Age at menopause[b] | ≧50<br>47.9% | <50<br>41.2% | 1.31 | $P = 0.1775$ (missing 170)[a] (NR[c] 250) |
| Familial breast cancer | Yes<br>43.4% | No<br>37.2% | 1.30 | $P = 0.1947$ (missing 3) |
| Oral contraceptive use | Ever<br>30.2% | Never<br>36.8% | 0.74 | $P = 0.1400$ (missing 139) |
| Breast complaints | Yes<br>53.6% | No<br>36.6% | 2.00 | $P = 0.0055$ (missing 4) |
| Breast aberrations in history[d] | Yes<br>36.1% | No<br>38.5% | 0.98 | $P = 0.6114$ (missing 4) |
| Wolfe classification | N1+P1<br>44.1% | P2+DY<br>32.4% | 1.65 | $P = 0.0007$ (missing 1) |

[a]The great number of missing data are due to the introduction in the second screening round of new blank forms, which excluded these questions; [b]Not relevant for women without children; [c]Not relevant for premenopausal women; [d]Aberrations: operation, mastitis, cyste or radiation in history.

**Figure 1** Distribution of the 787 true-positive and false-positive test result cases according to chance rates, predicted by the model:

$Pr = 1/(1 + \exp(-(4.005 + 0.060x_1 + 0.8398x_2)))$
Pr = chance of having a true-positive test result
$x_1$ = age at referral
$x_2$ = breast complaints (0 = no, 1 = yes)

(Hendriks, 1982; Verbeek, 1985). The sensitivity rates of the test, based on the occurrence rate of interval cancer in a 2-year observation period, are 45–60% for women under 50 but 60–80% for the older age group. Some of the interval cancers of the breast, however, may well have been non-existent at the time of this previous examination and may have grown rapidly. There are some indications that in women under the age of 50 a more aggressive kind of breast cancer occurs with a faster growth rate (Meyer *et al.*, 1984). If this is true a relatively great proportion of the interval cancers in this young age group will be newly developed cancers, and consequently the above-mentioned sensitivity rate of 45–60% would be too low. If the sensitivity and specificity rates are equal in both age groups, then the lower PV+ for women under 50 could only be the result of a lower prevalence of breast cancer in this age group.

In Table I an increase in PV+ is noticeable in round 5. It could be argued that this increase was influenced by the use of a new mammography apparatus in 1981. The PV+ for women under age 50 at referral did not change significantly: before 1982 the PV+ was 19.4% (43/222) on average, and after the replacement 31.0% (13/42); $P = 0.09$. For women aged 50 or over the PV+ was 43.7% (180/412) on average before 1982, and after it was 57.4% (66/115); $P < 0.01$.

However, a logistic regression analysis with 'yes/no referred via new apparatus' in the multivariate model, age included, did not yield a statistically significant result.

The PV+ in Nijmegen is high compared with other PV+ rates in the literature. In the HIP-study (Shapiro *et al.*, 1966) 111 women age 40–64 were referred to a surgeon at their first examination with mammography only. Twelve were diagnosed as having breast cancer (PV+ = 11%). Later reports on the HIP study show about a 20% biopsy positive rate for mammography. In the BCDDP projects a PV+ rate of 10–15% was estimated for women aged 35–74, who were referred for surgery (Baker, 1982). The Guildford Screening Project in England (Thomas *et al.*, 1983) invited women aged 45–64. In the first screening round the PV+ for surgical examination was 27.4% and the PV+ for biopsies was 36.1%. In a screening service in London (Chamberlain *et al.*, 1984) the PV+ for biopsies was low and decreased in the consecutive screening rounds from 14 to 5%, which could be the consequence of lower breast cancer prevalence (screening was conducted after ½ a year, 1 year and 2 years). In 1974 a screening project for women older than 40 was started in Sandviken, a city in Sweden (Andersson *et al.*, 1979). In the third screening round in 1980 the PV+ for clinical examination for women under age 50 was 37.5%. For women aged 50 or older the PV+ was 60.0%. Biopsy was performed in 27 women, 21 of whom proved to have breast cancer: the PV+ for biopsies was 78%. In 1976 a breast cancer screening project was started for women aged 50–69 in Malmö, Sweden (Lundgren & Helleberg, 1982). After a complete mammographic examination 211 women were referred for clinical examination: 45% proved to have breast cancer. In Kopparberg, another county in Sweden, a screening project started in 1977 (Tabár & Gad, 1981). Up to 1980, 1649 women, aged 39 or over, were referred for detailed mammographic examination and 362 underwent a biopsy. Cancer was diagnosed in 235 of them. The PV+ for referral was 14.3%, and the PV+ for biopsies was 65%. The DOM-project in Utrecht (de Waard *et al.*, 1984) estimated a PV+ of 40–57% for biopsies for women aged 50–64 in all screening rounds.

The various PV+ rates described in the literature are difficult to compare. The prevalence of breast cancer varies geographically and with age. Moreover, in some instances different screening intervals are used. Also different screening tests are used, e.g., some including physical examination as well. Viewing technique and mammographic equipment as well as experience and knowledge may vary with each project. In the US a more aggressive referral procedure maintains a high sensitivity, most likely at the expense of specificity and PV+. The result is that only 1 out of 10 or even 1 out of 20 women who undergo biopsy will prove to have breast cancer (Hall, 1986; Moskowitz & Gartside, 1982).

Another problem in comparing PV+ is the difference in referral procedures (Rombach, 1983). The percentage of women with breast cancer among referred women can be estimated at various stages in the general procedure: after single mediolateral oblique view, complete mammography, clinical examination, or surgical biopsy. The results presented in Table II, for instance, show different findings depending on whether the PV+ estimation is based on the numbers of women who were referred after single mediolateral oblique view or on that of women who had biopsy.

The increase in the biopsy PV+ in round five is large. As a consequence of the high PV+ the first results from the Nijmegen cancer registry show no high interval cancer rate after round five.

Women with true-positive and false-positive test results differ significantly in age and breast complaints. No difference was found in the prevalence of familial breast cancer, which could be due to the fact that the radiologist may already have considered this characteristic when he decided whether or not to refer the woman in case of hesitation. Women who have breast complaints at the time of an examination have, when referred, twice as high a chance of turning out true-positive cases as women without complaints do. One could argue that the screening examination came too late for these women since screening is supposed to detect breast cancer in an asymptomatic stage of the disease. Relatively few women, however, only 8% of the screened population, answered the question on breast complaints in the affirmative.

Although the groups of true-positive and false-positive cases differ significantly in age and breast complaints, these characteristics cannot be used to distinguish the two groups from each other as is shown in Figure 1, i.e., the specificity, which is very high as it is in Nijmegen, cannot be increased any further using the investigated characteristics in addition to mammographic results.

One could promote a more gradual referral procedure, where women contact a surgeon only in the last resort after a complete mammographic examination. In the first and second screening rounds in Nijmegen 23% of all women referred for clinical examination only received complete mammography showing no evidence of breast cancer after their referral; in the fifth screening round 16% did. This means that 16–23% of all referred women would not have contacted a surgeon in the gradual referral procedure where complete mammography would be performed after a suspect single oblique view. This procedure would be less of a strain on the patient and less expensive as well. One could then aim for a higher sensitivity of mammography for women under age 50, at the expense of specificity. Perhaps the lower specificity of the mammographic test in the gradual referral procedure could be improved by the use of some specific characteristics, in addition to mammographic results, in order to increase the overall specificity.

We thank H. Coopmans, Ms I. vd Steen (Department of Social Medicine) and Ms H. Rijken (Department of Radiology) for data analysis, processing, and gathering; and F de Groot for his comments. This study was supported by the Praeventiefonds.

### References

ANDERSSON, I., ANDRÉN, L., HILDELL, J., LINELL, F., LJUNGQVIST, U. & PETTERSSON, H. (1979). Breast cancer screening with mammography. *Diag. Radiol.*, **132**, 273.

BAKER, H.L. (1982). Breast Cancer Detection Demonstration Project: Five-year summary report. *CA*, **32**, 194.

BRESLOW, N.E. & DAY, N.E. (1980). Statistical methods in cancer research Vol. 1. The analysis of case-control studies. International Agency for Research on Cancer, Lyon, 162–248.

CHAMBERLAIN, J., CLIFFORD, R., NATHAN, B., PRICE, J.L. & BURN, I.A.N. (1984). Repeated screening for breast cancer. *J. Epidemiol. Community Hlth.*, **38**, 54.

COLLETTE, H.J.A., DAY, N.E., ROMBACH, J.J. & DE WAARD, F. (1984). Evaluation of screening for breast cancer in a non-randomised study (the DOM-project) by means of a case-control study. *Lancet*, **i**, 1224.

HALL, F.M.M.D. (1986). Screening mammography – Potential problems on the horizon. Sounding Board, *N. Engl. J. Med.*, **314**, 53.

HENDRIKS, J.H.C.L. (1982). Population screening for breast cancer by means of mammography in Nijmegen 1975–1980. Koninklijke drukkerij G.J. Thieme bv, Nijmegen.

LUNDGREN, B. & HELLEBERG, A. (1982). Single oblique-view mammography for periodic screening for breast cancer in women. *J. Natl Cancer Inst.*, **68**, 351.

MEYER, J.S., McDIVITT, R.W., STONE, K.R., PREY, M.U. & BAUER, W.C. (1984). Practical breast carcinoma cell kinetics: Review and update. *Breast Cancer Res. Treat.*, **4**, 79.

MOSKOWITZ, M. & GARTSIDE, P.S. (1982). Evidence of breast cancer mortality reduction: Aggressive screening in women under age 50. *Amer. J. Radiol.*, **138**, 911.

ROMBACH, J.J. (1983). Periodic breast cancer screening with single oblique-view mammography. *J. Natl Cancer Inst.*, **70**, 1. (*Letter*).

SHAPIRO, S., STRAX, PH. & VENET, L. (1966). Evaluation of periodic breast cancer screening with mammography. Methodology and early observations. *J. Amer. Med. Assoc.*, **195**, 731.

SHAPIRO, S., VENET, W., STRAX, PH., VENET, L. & ROESER, R. (1982). Ten- to fourteen-year effect of screening on breast cancer mortality. *J. Natl Cancer Inst.*, **69**, 349.

TABÁR, L. & GAD, A. (1981). Screening for breast cancer: The Swedish Trial. *Radiology*, **138**, 219.

TABÁR, L., FAGERBERG, C.J.G., GAD, A. & 9 others (1985). Reduction in mortality from breast cancer after mass screening with mammography. *Lancet*, **i**, 829.

THOMAS, B.A., PRICE, J.L. & BOULTER, P.S. (1983). The Guilford Breast Screening Project. *Clin. Oncol.*, **9**, 121.

VERBEEK, A.L.M., HENDRIKS, J.H.C.L., HOLLAND, R., MRAVUNAC, M., STURMANS, F. & DAY, N.E. (1984). Reduction of breast cancer mortality through mass screening with modern mammography: First results of the Nijmegen Project, 1975–1981. *Lancet*, **i**, 1222.

VERBEEK, A.L.M. (1985). Population screening for breast cancer in Nijmegen. An evaluation of the period 1975–1982. Instituut Sociale Geneeskunde Katholieke Universiteit, Nijmegen.

DE WAARD, F., COLLETTE, H.J.A., ROMBACH, J.J., BAANDERS-VAN HALEWIJN, E.A. & HONIG, C. (1984). The DOM project for the early detection of breast cancer, Utrecht, The Netherlands. *J. Chron. Dis.*, **37**, 1.

WOLFE, J.N. (1976). Breast patterns as an index of risk for developing breast cancer. *Am. J. Roentgenol*, **126**, 1130.